# Cultivar-Dependent Differences in Agronomic Characteristics, Nutritional Value, Fermentation Quality, and Bacteriome Profile of Whole-Plant Sorghum Silage

**DOI:** 10.3390/microorganisms13112634

**Published:** 2025-11-20

**Authors:** Yawei Zhang, Danqi Feng, Juanli Huo, Jiabao Xu, Yuehong Wang, Qiang Liu, Wenbin Bai, Qingshan Liu, Yuanqing Zhang

**Affiliations:** 1 College of Animal Science, Shanxi Agricultural University, Taiyuan 030801, China; ywzhang@sxau.edu.cn (Y.Z.);; 2 Sorghum Research Institute, Shanxi Agricultural University, Jinzhong 030600, China

**Keywords:** agronomic traits, forage sorghum, grain sorghum, lactic acid bacteria, *Leuconostoc*, silage microbiome

## Abstract

Forage scarcity in semi-arid regions necessitates the identification of optimal sorghum cultivars for high-quality silage production. This study systematically evaluated varietal differences in agronomic characteristics, nutritive value, fermentation quality, and bacterial community structure of whole-plant sorghum silage. A completely randomized design was implemented with four sorghum cultivars representative of semi-arid northwestern China: Liaotian1 (LT1), Jinnuo3 (JN3), Jinza2001 (JZ2001), and Jinza1531 (JZ1531). Five quadrats per cultivar in experimental fields were randomly designated as biological replicates for silage production. The plants were harvested at the dough stage, chopped, and ensiled in laboratory-scale silos (*n* = 20, 4 cultivars × 5 replicates) for 120 days. Analyses included agronomic measurements, chemical composition, fermentation parameters, microbial plate enumeration, and bacterial community profiling via 16S rRNA gene amplicon sequencing of the V3–V4 hypervariable region. The results showed that cultivar significantly influenced (*p* < 0.01) all agronomic traits and most nutritional parameters. The forage-type cultivar LT1 showed the highest biomass yield but the lowest nutritional quality, with higher neutral detergent fiber (47.77% vs. 29.21–32.35%; *p* < 0.05) and lower starch (10.94% vs. 18.10–24.30%; *p* < 0.05) contents as well as higher dry matter losses (1.39% vs. 0.91–1.23%; *p* < 0.05) than grain-type cultivars. In contrast, the grain-type cultivar JN3 exhibited balanced yield-quality traits with the highest (*p* < 0.05) starch (24.30%) and crude protein (7.50%) contents. Most fermentation parameters differed significantly (*p* < 0.01) among cultivars, with JN3 showing elevated ammonia-nitrogen (0.24 g/kg) but within acceptable ranges. Microbial diversity analysis revealed cultivar-driven differences in bacterial communities, with JN3 enriched in *Leuconostoc* and early-colonizing taxa (*p* < 0.05 and LDA Score > 4). It is concluded that the grain-type cultivar JN3 is the most suitable cultivar for whole-plant sorghum silage production in water-limited regions due to its optimal yield-quality balance. The findings underscore the importance of integrated cultivar evaluation and suggest the potential of targeted microbial inoculants for enhancing silage quality.

## 1. Introduction

Sorghum (*Sorghum bicolor*), ranked as the fifth most cultivated cereal crop worldwide after maize, wheat, rice, and barley, is a critical resource for food security, animal feed, brewing industry, and sustainable biofuel production [1,2,3]. As a C4 crop, sorghum demonstrates superior agronomic productivity and exceptional tolerance to multiple abiotic stresses including drought, salinity, and elevated temperatures, attributed to its extensive root architecture and efficient C4 photosynthesis pathway [4]. These adaptive traits enable sorghum to maintain high yields across diverse environments, including marginal lands with poor soil quality [3]. Given the dual challenges of global climate change and food security, sorghum has consequently emerged as a promising climate-resilient alternative crop for the food–feed–bioenergy multipurpose, particularly in water-limited arid and semi-arid regions [5,6,7]. Furthermore, the emergence and spread of the Western corn rootworm have prompted sorghum adoption as a viable rotational alternative to corn in affected regions [4].

Cultivated varieties of sorghum exhibit remarkable phenotypic and morphological diversity, reflecting its distinct domestication histories across geographic regions. Based upon the usage and production characteristics (such as grain yield, seed size, number of tillers, stem shape, weight, sugar content, etc.), these have been classified into three broad types namely: grain, forage, and energy sorghum [2,7,8]. Grain-type sorghum varieties are characterized by their large panicles and are primarily used for human consumption, brewing, and livestock feed. Forage-type sorghum varieties are distinguished by their rapid growth rate and high biomass yield, and are predominantly cultivated for silage, hay production, and pasture grazing. The forage sorghums are further grouped into four types: (1) hybrid forage sorghum, (2) Sudangrass, (3) Sorghum × Sudangrass hybrids, and (4) sweet sorghum [2]. Energy-type sorghum cultivars are bred for elevated non-structural carbohydrate accumulation and enhanced lignocellulosic biomass production, making it an ideal feedstock for bioethanol and advanced biofuels production [8]. Sweet sorghum is one of the leading sorghum varieties for biofuel feedstock. It is notable that variation can be observed both physically and chemically between sorghum varieties and hybrids [7].

Although serving as a source of staple food for over 500 million people across more than 30 nations in Africa and Asia, sorghum is predominantly utilized as livestock feed, accounting for approximately 62% of global production [3]. This utilization pattern is typified by ensiling, an anaerobic fermentation process mediated by lactic acid bacteria (LAB), which has become principal preservation strategy for all sorghum varieties in modern animal husbandry, especially ruminant production systems [5]. Ensiling represents a complex microbiological process where the composition and activity of the epiphytic microbiome—particularly the bacteriome—are critical determinants of fermentation profile and final silage quality. Specifically, the relative abundance of LAB versus spoilage-associated bacteria directly influences the rate of acidification, extent of dry matter preservation, and final nutritional value [9]. Variation in chemical composition among sorghum varieties may significantly impact both the establishment of silage microbiome and the ensuing fermentation dynamics, thereby affecting subsequent nutritive value and animal performance. However, there is lack of information about which type of sorghum varieties should be preferentially used for production of sorghum silage in ruminant feeding.

Accordingly, this study aimed to identify optimal sorghum cultivars for whole-plant silage production in semi-arid northwestern China through comprehensive evaluation of the varietal differences in pre-ensiling agronomic performance and post-ensiling quality parameters, including chemical composition, nutritive value, fermentation profile, microbial viability, and bacteriome diversity. Four representative sorghum cultivars from northwestern China were investigated: (1) Liaotian 1 (LT1), a forage-type sweet sorghum representing high-biomass genotypes bred specifically for silage production; (2) Jinnuo3 (JN3), a grain-type waxy cultivar characterized by its high grain yield and starch content; (3) Jinza 2001 (JZ2001), a grain-type non-waxy hybrid, a widely adapted commercial cultivar valued for its stress tolerance; and (4) Jinza 1531 (JZ1531), a grain-type non-waxy hybrid known for its stable yield. These cultivars were strategically selected to represent the genetic and phenotypic diversity of commercially relevant sorghum types, encompassing distinct breeding targets and end-use applications. We hypothesized that cultivar-specific genetic backgrounds would drive chemical composition differences that subsequently shape microbiome assembly during ensiling, ultimately determining fermentation profile and silage quality. Specifically, we postulated that forage-type cultivar would maximize biomass production at the expense of nutritional density, while grain-type cultivars would provide better feeding value through superior starch and protein accumulation.

## 2. Materials and Methods

### 2.1. Cultivation, Harvest, and Ensiling of Sorghum Cultivars

Four sorghum cultivars—LT1, JN3, JZ2001, and JZ1531—were cultivated in experimental fields at the Sorghum Research Institute of Shanxi Agricultural University (112°71′ E, 37°59′ N) in northwestern China during the 2021 growing season (June–October). The region experiences a typical warm temperate semi-humid continental monsoon climate, characterized by distinct seasonal variations. Meteorological data from the 2021 growing season recorded a mean annual temperature of 9.6 °C, average annual precipitation of 456.8 mm, the annual sunshine hours are 2662 h, and the frost-free period is 158 days. The area exhibits significant intra-annual variation in precipitation distribution, with approximately 70% of rainfall occurring during the summer months (June–August). Additional climatic parameters included an annual evaporation rate of 1782.4 mm and average relative humidity of 64%, creating a water-deficient environment typical of semi-arid agricultural regions. The experimental field featured Calcaric Cinnamon soil with medium loam texture. Analysis of the topsoil layer (0–25 cm) indicated the following characteristics: pH 7.6, organic matter 12.7 g/kg, total nitrogen 0.98 g/kg, available phosphorus 12.45 mg/kg, and available potassium 101.9 mg/kg. Each cultivar was cultivated in experimental plots measuring 25 m × 100 m, with a planting configuration of 50 cm row spacing, 20 cm plant spacing, and a density of 100,000 plants per hectare. Prior to planting, a basal fertilizer application was implemented, consisting of 18.9 t/ha of organic fertilizer and 750 kg/ha of chemical complex fertilizer. The chemical complex fertilizer (Stanley Fertilizer Co., Ltd., Chengde City, China) provided balanced nutrient content with 17% N, 17% P_2_O_5_, and 17% K_2_O. The organic amendment (Hebei Rundong Fertilizer Co., Ltd., Zhengding County, Hebei, China) comprised cow manure with an air-dried composition of 41.5% organic matter, 1.67% N, 0.43% P_2_O_5_, and 0.95% K_2_O. All field management practices, including irrigation scheduling, pest control measures, and weed management, followed established local agricultural protocols.

All four sorghum cultivars were harvested at the dough stage on 17 October 2021. For each cultivar, whole plants were manually harvested at 4–6 cm above ground level from 5 randomly representative quadrats (2 m × 3 m) within the experimental plots. Agronomic traits, including plant height (ground level to panicle top), ear height (ground level to panicle base), leaf counts, whole-plant fresh weight, and panicle weight, were measured immediately after harvest on 20 randomly selected plants per quadrate for each cultivar. Whole-plant fresh yield (t/ha) was estimated by multiplying the fresh weight of all plants per quadrat by the ratio of hectare to quadrat area.

Following transportation to the Sorghum Research Institute, the whole plant sorghum from each quadrat of each cultivar was uniformly chopped into approximately 2 cm length using a straw breaker (Model KJ-400, Kunjieyucheng Machinery Equipment Co., Beijing, China). The chopped plant material from each cultivar was separately packed into 5 L laboratory-scale silos. All silos were compacted to a target bulk density of 878.8 ± 14.8 kg/m^3^ (fresh weight basis), with achieved densities of 881.6 ± 10.9 kg/m^3^ (LT1), 875.2 ± 12.4 kg/m^3^ (JZ1531), 881.5 ± 11.7 kg/m^3^ (JN3), and 876.8 ± 13.2 kg/m^3^ (JZ2001). Statistical analysis confirmed no significant differences in compaction density among cultivars, ensuring uniform oxygen exclusion and consistent fermentation conditions across all experimental units. The polyethylene silos were fitted with threaded caps containing butyl rubber gaskets to ensure anaerobic conditions. Parallel to silo preparation, representative fresh samples from each cultivar quadrate were immediately prepared for analysis of dry matter (DM) at harvest. DM yield per hectare was calculated based on fresh weight each cultivar quadrate and corresponding DM content. In total, 20 experimental silos (5 quadrates × 4 cultivars) containing whole-plant sorghum silage were prepared and transferred to the College of Animal Science at Shanxi Agricultural University for storage. During the 120-day storage period, silos were randomly arranged in a dedicated storage facility and maintained under ambient conditions. Ambient temperature was monitored daily using multi-channel data loggers (SMOWO MDL-1048A, Shanghai Tianhe Automation Instrument Co., Ltd., Shanghai, China), which confirmed a stable temperature range of 15–25 °C throughout the ensiling period. This controlled storage environment ensured consistent fermentation conditions across all experimental units. Afterwards, they were opened and sampled for analyses of chemical composition, fermentation characteristics, microbial enumeration, and bacteriome diversity. Meanwhile, each experimental silo was weighed (Model YHW-L01, Huizhou Yingheng Electronic Technology Co., Ltd., Huizhou, China) immediately post-sealing and pre-opening at 120 days, and its DM loss during ensiling was calculated gravimetrically based on fresh weight and corresponding DM content.

### 2.2. Chemical Composition Analysis and Estimation of Nutritive Feed Value

Following silo unsealing, the upper 5 cm layer of silage in each experimental silo was aseptically discarded to eliminate potential aerobic spoilage, and the remaining material was then mixed well on a clean bench prior to subsampling. From each silo, approximately 200 g silage were randomly collected in duplicate, yielding a total of 40 samples (4 cultivars × 5 biological replicates × 2 technical duplicates) for chemical composition analysis.

All samples were dried to constant weight in a forced-air oven at 65 °C for 48 h for DM determination, then ground using a laboratory fodder grinder (Model KRT-34, Kunjieyucheng Machinery Equipment Co., Beijing, China) equipped with a 1 mm stainless steel sieve. Afterwards, proximate composition analysis was performed on air-dried samples following Association of Official Analytical Chemists standard methods [10]: absolute DM (no. 934.01), crude protein (CP, no. 976.05), ether extract (EE, no. 920.39), neutral detergent fiber (NDF, no. 2002.04), acid detergent fiber (ADF, no. 973.18) and lignin (ADL, no. 973.18), ash (no. 942.05), calcium (Ca, no. 927.02), and phosphorus (P, no. 965.17) contents. Specifically, absolute DM was determined by oven-drying at 105 °C for 4 h. Nitrogen (N) content was determined via the Kjeldahl method using a semi-automatic analyzer (Kjeltec 8400, Foss Analytics, Hillerød, Denmark), with CP calculated as N × 6.25. The EE analysis was performed using an automatic fat analyzer (XT10, ANKOM Technologies, Macedon, NY, USA). The NDF and ADF were analyzed with an automatic fiber analyzer (A2000i, ANKOM Technologies, Macedon, NY, USA), with the use of sodium sulfite and heat-stable a-amylase in the NDF assay. All fiber fraction values were reported on an ash-inclusive basis. Calcium and phosphorus contents were determined using potassium permanganate titration and a UV spectrophotometer (UV-VIS 8500, Shanghai Tianmei Scientific Instrument Co., Ltd., Shanghai, China), respectively. Gross energy (GE) was quantified using an adiabatic oxygen bomb calorimeter (ZDHW-8, Hebi Tianke Coal Quality Instrument Co., Ltd., Hebi City, China), with benzoic acid (99.9% purity) as the calibration standard. Starch content was determined enzymatically following the protocol as described by Xiong et al. [11]. Tannin content was analyzed spectrophotometrically (λ = 525 nm) as described by Dalby and Shuman [12]. Chemical composition data was corrected for unpreventable volatile losses during drying using the measured total volatile fatty acids (TVFA) content. Non-fiber carbohydrates (NFC) content was calculated by difference as %NFC = 100 − (%CP + %NDF + %EE + %Ash). Relative feed value (RFV) was calculated using the equations described by Rohweder et al. [13] and Seglar and Shaver [14] as follows:

RFV = (DMI × DDM)/1.29

where DMI = 120/NDF and DDM = 88.9 − (0.779 × ADF). DMI represents the estimated dry matter intake and DDM denotes the digestible dry matter content of whole-plant sorghum silage. All fiber values (NDF and ADF) are expressed as % dry matter.

Total digestible nutrients (TDN) were estimated using the equation [15] as follows:

TDN% = 87.84 − (0.70 × ADF%)

### 2.3. Analysis of Fermentation Profile and Microbial Enumeration

Immediately following silo unsealing and homogenization, duplicate 30 g subsamples were collected from each experimental unit for fermentation profile analysis. Each silage sample (30 g) was mixed with 270 mL distilled water (1:9 *w*/*v*), and then blended for 60 s using a high-speed blender (WBL2521H, Midea Life Appliance Manufacturing Co., Ltd., Foshan, China). The slurry was sequentially filtrated through four layers of sterile cheesecloth followed by a 0.22 μm pore-size polyethersulfone membrane filters. The filtrate pH was immediately determined using a calibrated glass-electrode pH meter (PHS-3C, INESA Scientific Instrument Co., Ltd., Shanghai, China). Afterwards, organic acid profiles (lactate, formate, acetate, butyrate, and valerate) in the filtrate were analyzed by high-performance liquid chromatography (HPLC, 1260 Infinity II system, Agilent Technologies, Waldbronn, Germany) fitted with Hi-Plex H column (300 mm × 7.7 mm) and G7114A UV-detector, following the chromatographic conditions described by Zhang et al. [16]. Ammoniacal nitrogen (NH_3_-N) concentration in the filtrate was quantified using phenol-sodium hypochlorite colorimetry according to Broderick and Kang [17], with absorbance measured at 630 nm.

For microbial enumeration, another 20 g subsamples each bucket silo were aseptically collected immediately post-homogenization, and then extracted with 180 mL of sterile physiological saline at 4 °C, 150 rpm for 2 h using an orbital shaker incubator (SPX-250B-D, Boxun Industry Co., Ltd., Shanghai, China). The resulting supernatant was serially diluted (10^−1^ to 10^−6^) in sterile normal saline. LAB, coliforms, yeast, and molds were enumerated via plate counting on selective media as described by Zhang et al. [16].

### 2.4. Microbial Diversity Analysis

Approximately 20 g subsamples were aseptically collected from each experimental silo immediately post-homogenization, flash-frozen in liquid nitrogen, and store at −80 °C pending genomic DNA extraction for bacterial diversity analysis. Total genomic DNA was extracted individually from all 20 samples using the MagAtrract PowerSoil Pro DNA Kit (Qiagen, Hilden, Germany) following the bead-beating protocol [18]. The yield and purity of extracted DNA were quantified using a NanoDrop 2000 spectrophotometer (Thermo Scientific, Wilmington, NC, USA), while their integrity was checked by 1% agarose gels electrophoresis. Quality-verified DNA was employed as template to amplify the V3–V4 hypervariable region of bacterial 16S rRNA genes with the universal primer pairs 338F (5′-ACTCCTACGGGAGGCAGCAG-3′) and 806R (5′-GGACTACHVGGGTWTCTAAT-3′). PCR reaction solution (20 μL total volume) consisted of 2 × Pro Taq 10 μL, F/R primer (5 μM) 0.8 μL each, template DNA 10 ng, and finally ddH_2_O replenishment. Amplification was performed in a GeneAmp^®^ 9700 thermocycler (Applied Biosystems, Foster City, CA, USA) under the following conditions: initial denaturation at 95 °C for 3 min, followed by 29 temperature control cycles (denaturation at 95 °C for 30 s, annealing at 53 °C for 30 s, extension at 72 °C for 45 s), final extension at 72 °C for 10 min, and hold at 10 °C. All samples were amplified in triplicate, and the resulting PCR products were pooled prior to downstream processing. Amplicons were checked and enriched by 2% agarose gel electrophoresis, purified using the AxyPrep DNA Gel Extraction Kit (Axygen Biosciences, Union City, CA, USA) according to manufacturer’s instructions, and then quantified via fluorometry (Quantus^TM^, Promega Corporation, Madison, WI, USA). Equimolar amounts of purified amplicons were combined for library establishment using the NEXTflex^TM^ rapid DNA-seq kit (Bioo Scientific, Austin, TX, USA) following the merchandise instructions. Final libraries were paired-end sequenced (2 × 300 bp) on an Illumina MiSeq PE300 platform (Illumina Inc., San Diego, CA, USA) by Majorbio Bio-Pharm Technology Co., Ltd. (Shanghai, China), following standard manufacturer protocols.

Off-line sequencing data was demultiplexed, quality-filtered, and assigned to samples based on their unique barcode by Fastp (version 0.20.0) [19]. The demultiplexed paired reads were imported into the platform of QIIME2 (version 2022.08) [20] for subsequently processing and analysis following a modified version of pipeline as previous described [16]. Briefly, the barcode and primer sequences were removed from the raw paired-end reads via the trim-paired method integrated in the q2-cutadapt plugin [21]. The generated paired reads were quality-filtered, merged, dereplicated, and chimera-removal using the denoise-paired method integrated in the q2-dada2 plugin to obtain amplicon sequence variants (ASVs) with abundance table [22]. This process was performed by truncating forward and reverse reads at position 266 and 184 bases, respectively. Taxonomic assignment of the ASVs was performed as described by Zhang et al. [16].

In order to analyze phylogenetic diversity (PD), a maximum-likelihood phylogenetic tree was constructed using the align-to-tree-mafft-fasttree pipeline integrated in the q2-phylogeny plugin. To enable cross-sample comparisons, sequence counts were rarefied to an even depth of 48,236 sequences per sample using the rarefy command of the q2-feature-table plugin, representing the minimum sequencing depth across all samples. The phylogenetic tree and the rarefied feature table were then used to calculate the alpha diversity indices, including Shannon entropy, observed features, Faith PD, and Pielous evenness, and beta diversity indices, including unweighted UniFrac and weighted UniFrac distance, using the core-metrics-phylogenetic pipeline from the q2-diversity plugin. In addition, sequencing saturation was assessed through alpha rarefaction curves plotting Shannon entropy and Faith’s PD metrics against sequencing effort using the rarefy command.

### 2.5. Statistical Analysis

Microbial enumeration data were log10-transformed to meet normality assumptions prior to statistical analysis. The data of agronomic characteristics, chemical composition, fermentation parameters, and transformed microbial counts were analyzed using the GLM procedure of SAS software (version 9.4, SAS Institute Inc., Cary, NC, USA), with the following general linear model:

Y_i_ = μ + T_i_ + ε_i_

where Y_i_ is the observed value, μ is the overall mean, T_i_ is the fixed effect of the ith sorghum cultivar (i = LT1, JN3, JZ2001, JZ1531), and ε_i_ is the random residual error. Post hoc multiple comparisons were performed using Duncan’s test, with statistical significance defined at *p* < 0.05. Data are presented as least-square means and standard error of the mean (SEM).

Bacterial alpha diversity differences among sorghum cultivars were checked using the Kruskal–Wallis test through the alpha-group-significance command from the q2-diversity plugin. Beta diversity patterns were visualized via principal coordinates analysis (PCoA) of both Unweighted UniFrac and Weighted UniFrac distance at the ASVs level, implemented through the qiime2R package [23] in R (v4.1.2). Statistical significance of community composition differences among cultivars was evaluated using permutational multivariate analysis of variance (PERMANOVA; 999 permutations) with the beta-group-significance command of the q2-diversity plugin.

Bacterial composition profiles were analyzed at both phylum and genus taxonomic levels. For each level, we established two classification thresholds: (1) the taxa with relative abundance > 0.01% in at least one sample as identified, and (2) the taxa with relative abundance > 0.01% and presented in more than half samples as detected. Only detected taxa were used for downstream statistical analysis. Relative abundances were arcsine square-rooted transformed and then compared across cultivars using GLM procedure as aforementioned. To further identify differentially abundant taxa, a more stringent linear discriminant analysis (LDA) effect size (LEfSe) analysis [24] was performed, and taxa with LDA score > 2 and *p* < 0.05 were considered as significantly different.

## 3. Results

### 3.1. Agronomic Traits of Four Sorghum Cultivars

The agronomic characteristics covering plant height, ear height, leaf number, fresh plant weight, flesh ear weight, panicle proportion, fresh yield, and DM yield of the four sorghum cultivars are presented in Table 1. It can be seen from the table that all measured agronomic traits exhibited significant cultivar effects (*p* < 0.01). Among the tested cultivars, LT1 displayed the greatest plant height, ear height, leaf number, and fresh plant weight (*p* < 0.05), while concurrently exhibiting the lowest fresh ear weight and panicle proportion (*p* < 0.05). In contrast, JZ2001 had the lowest plant height, ear height, and fresh plant weight (*p* < 0.05), but had the greatest panicle proportion of whole-plant (*p* < 0.05). JN3 and JZ1531 exhibited similar intermediate values for plant height, ear height, leaf number, and panicle proportion of whole-plant, while concurrently displaying the greatest fresh ear weight (*p* < 0.05).

In terms of yield performance, cultivar LT1 demonstrated superior yield, producing the highest (*p* < 0.05) fresh whole-plant (118.95 t/ha) and DM (32.91 t/ha) yields. Yields for JN3 (75.75 and 25.77 t/ha, respectively) and JZ1531 (70.35 and 28.14 t/ha, respectively) were statistically similar but significantly lower than LT1 (*p* < 0.05). JZ2001 recorded the lowest (*p* < 0.05) values (44.55 and 18.29 t/ha for fresh and DM yield, respectively).

### 3.2. Chemical Composition and Nutritive Value

Table 2 shows the chemical composition and nutritive value of four cultivars of whole-plant sorghum silage. Statistical analysis revealed that variety had a significant effect on DM losses, total digestible nutrients, relative feed value, and concentrations of DM, starch, crude protein, ether extract, neutral detergent fiber, acid detergent fiber, acid detergent lignin, tannin, and phosphorus (*p* < 0.01), but did not affect the concentration of gross energy, ash, or calcium of whole-plant sorghum silage (*p* > 0.05).

LT1 had the highest DM loss during ensiling (1.39%, *p* < 0.05), significantly exceeding JZ1531 (1.23%, *p* < 0.05), whereas JN3 (0.92%) and JZ2001 (0.91%) exhibited the lowest DM losses (*p* < 0.05). Regarding the chemical composition of whole-plant sorghum silage from various cultivars, DM content was lower (*p* < 0.05) in LT1 (24.85%) than that in JZ1531 (38.83%), JZ2001 (38.56%), and JN3 (37.96%). The concentrations of both starch and crude protein were highest in cultivar JN3 (*p* < 0.05), followed by JZ2001 (*p* < 0.05), and then JZ1531 (*p* < 0.05), whereas LT1 showed the lowest levels for both components (*p* < 0.05). In contrast, LT1 silage exhibited the highest NDF and ADF contents (*p* < 0.05), which were significantly greater than those in JZ1531 (*p* < 0.05), while JN3 and JZ2001 recorded the lowest NDF and ADF concentration (*p* < 0.05). Consistent with this trend, LT1 also had the highest ADL content (*p* < 0.05), followed by JZ1531 and JZ2001 (*p* < 0.05), while JN3 ranked lowest (*p* < 0.05). Ether extract content was highest in JZ2001 (1.68%) and lowest in LT1 (0.91%), both were significantly (*p* < 0.05) different from JN3 (1.47%) and JZ2001 (1.51%). Phosphorus levels were significantly highest in JZ2001, followed by JZ1531 and then JN3, whereas LT1 had the lowest phosphorus content. In addition, the significantly highest tannin levels (*p* < 0.05) were observed in JZ2001 (0.154%) and JZ1531 (0.178%), followed by JN3 (0.096%, *p* < 0.05), whereas LT1 showed the lowest level (0.050%, *p* < 0.05).

Regarding nutritional quality, both TDN and RFV were significantly highest in JN3 and JZ2001 (*p* < 0.05), followed by JZ1531 (*p* < 0.05), while LT1 consistently showed the lowest values (*p* < 0.05).

### 3.3. Fermentation Parameters and Microbial Counts

The fermentation parameters and microbial counts data in whole-plant sorghum silage with different varieties are presented in Table 3. Statistical analysis results demonstrated significant cultivar effects (*p* < 0.01) on the fermentation parameters of whole-plant sorghum silage, with the exception of acetic acid content (*p* = 0.31). However, no significant varietal differences (*p* > 0.05) were observed in the enumeration of predominant silage microbiota, including LAB, coliform bacteria, yeasts, and molds.

The whole-plant sorghum silage from LT1 showed a significantly lower (*p* < 0.05) pH value (3.99) compared to the other three cultivars (JN3, 4.12; JZ2001, 4.15; JZ1531, 4.11). Ammonia nitrogen (NH_3_-N) content was highest in JN3 (*p* < 0.05), followed by JZ2001 (*p* < 0.05), with LT1 and JZ1531 showing the lowest values (*p* < 0.05). Notably, the NH_3_-N/TN ratio was highest in LT1 (*p* < 0.05), followed by JN3 (*p* < 0.05), whereas JZ2001 and JZ1531 ranked lowest (*p* < 0.05). With regard to the organic acid profiles, JZ2001 and JZ1531 produced the highest lactate concentration (*p* < 0.05), followed by JN3 (*p* < 0.05), while LT1 showed the lowest (*p* < 0.05). Formate content was substantially higher (*p* < 0.05) in JN3 (1.48 g/kg) and LT1 (1.51 g/kg) compared to JZ2001 (0.74 g/kg) and JZ1531 (0.60 g/kg). Butyrate concentration was highest in JN3 (*p* < 0.05), followed by JZ2001 (*p* < 0.05), with LT1 and JZ1531 exhibiting the lowest values (*p* < 0.05). Similarly, valerate content was also highest in JN3 (*p* < 0.05), followed by LT1 (*p* < 0.05), whereas JZ2001 and JZ1531 showing the lowest values (*p* < 0.05). Consequently, both the total organic acids concentration and the lactate-to-acetate ratio were significantly diminished in LT1 relative to the other three cultivars (*p* < 0.05).

### 3.4. Bacteriome Diversity

In the present study, a total of 1,234,648 pair-end reads were generated in the amplicon sequencing, with an average of 64,981 ± 4232 (*n* = 20) pair-end reads per sample. After quality control and denoising, including primer removal, reads merging, and chimera filtering, a total of 1,078,098 (56,742 ± 3750; *n* = 20) high-quality sequences were obtained, with 1731 amplicon sequence variations (ASVs) averaging 406 ± 44 bases in length. The sequencing index good’s coverage for each sample was all above 0.9999, while the rarefaction curves based on Faith PD and Shannon entropy for each sample level out as the sampling depth outnumbered 18,000 (Supplementary Figure S1), indicating that the sequencing depth was deep enough to cover the entire bacterial community, assuring the representativeness of sequencing data.

**Figure 1:**
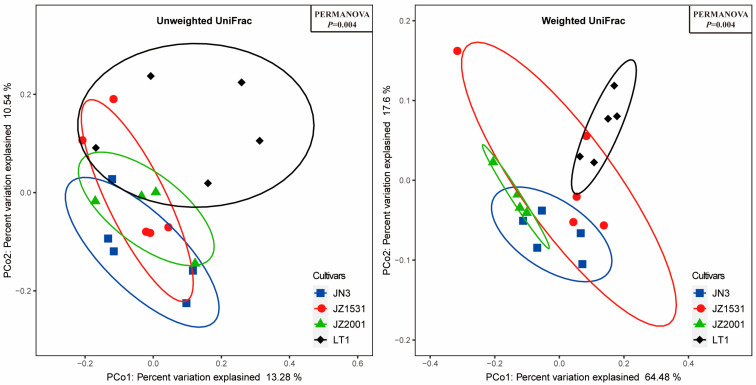
Dissimilarity of the bacterial profiles in the whole-plant sorghum silage across cultivars. Distance between samples based on similarity in ASVs composition was calculated using unweighted UniFrac (**left**) and weighted UniFrac distance (**right**), and visualized using PCoA plots. The impact of variety on the clustering pattern of bacterial communities was tested using PERMANOVA. The ovals in varied colors represent 95% confidence interval of silage bacterial profiles for each cultivar.

Variations in the alpha and beta diversity of bacterial microbiota in whole-plant sorghum silage across four cultivars were demonstrated in Table 4 and Figure 1, respectively. The alpha diversity analysis revealed significant cultivar-dependent variations (*p* < 0.04) in Shannon entropy, observed features, and Pielou’s evenness, while Faith’s PD showed no significant differences (*p* = 0.23) among cultivars. Post hoc analysis showed that JN3 exhibited significantly greater observed features and Shannon entropy of bacterial community than both JZ2001 and JZ1531 (*p* < 0.05), while LT1 showing intermediate values that did not differ significantly from any other cultivar (*p* > 0.05). Similarly, Pielou’s evenness was higher in JN3 silage than that in the other three cultivars (*p* < 0.05). Principal coordinate analysis based on either unweighted UniFrac or weighted UniFrac metrics revealed that the samples from each cultivar were individually clustered within a narrow range, with the confidence ellipses of each cultivar tending to separate from each other (Figure 1). PERMNOVA results substantiated significant compositional dissimilarities in bacterial communities across cultivars (*p* = 0.004), though the bacterial profile in JZ1531 silage did not differ statistically from that of either JN3 or JZ2001 silages (*p* > 0.05).

**Figure 2:**
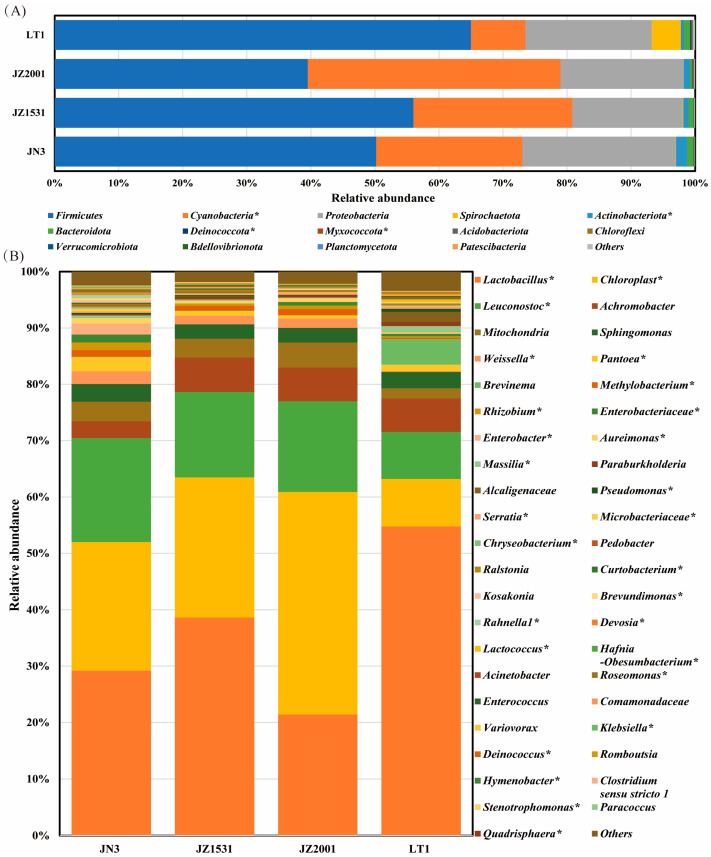
Bacterial composition at the phylum (**A**) and genus (**B**) level in the whole-plant sorghum silage across cultivars. Taxa name with asterisk (*) denoted significant cultivar effect (*p* < 0.05).

Taxonomic classification revealed a total of 30 and 113 bacterial taxa at the phylum and genus level as being identified, 14 and 43 of which were classified as being detected according to the cutoff defined in Section 2 “Materials and Methods”. The detected bacterial taxa at both phylum and genus levels in the whole-plant sorghum silage across cultivars were illustrated in Figure 2 and Supplementary Table S1. The top five abundant phyla were *Firmicutes*, *Cyanobacteria*, *Proteobacteria*, *Spirochaetota*, and *Actinobacteriota*, with an average relative abundance of 53.38%, 23.08%, 20.06%, 1.25%, and 0.99%, respectively. Intergroup differences analysis revealed that the relative abundance of four phyla—*Cyanobacteria*, *Actinobacteriota*, *Deinococcota*, and *Myxococcota*—were significantly influenced by sorghum cultivar (*p* < 0.05). At the genus level, the top three dominant taxa were *Lactobacillus* (36.76%), *Chloroplast* (23.07%), and *Leuconostoc* (14.42%). Our results demonstrated the cultivars significantly influenced (*p* < 0.05) the relative abundance of 27 bacterial genera in whole-plant sorghum silage, particularly affecting key LAB genera including *Lactobacillus*, *Leuconostoc*, *Weissella*, and *Lactococcus*.

**Figure 3:**
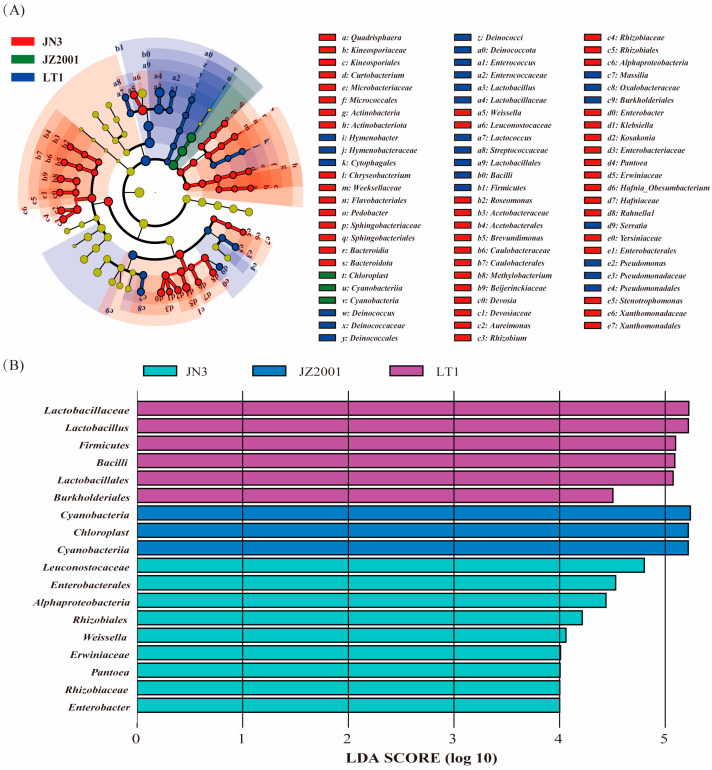
Cladogram ((**A**): *p* < 0.05 and LDA Score > 2) and histogram ((**B**): *p* < 0.05 and LDA Score > 4) representation of phylogenetically discriminative silage bacterial taxa identified by Linear Discriminant Analysis Effect Size (LEfSe) analysis.

LEfSe analysis revealed a significant difference (*p* < 0.05 and LDA Score > 2) in the relative abundance of 72 detected bacterial taxa in whole-plant sorghum silage among cultivars (Figure 3A). Refined LEfSe analysis with stricter thresholds (*p* < 0.05 and LDA Score > 4) revealed 18 bacterial taxa showing significant associations with sorghum cultivars, with LT1 silage exhibiting the highest abundance of *Firmicutes*, *Bacilli*, *Lactobacillales*, *Lactobacillaceae*, *Lactobacillus*, and *Burkholderiales*, JZ2001 silage exhibiting the highest abundance of *Cyanobacteria* and *Chloroplast*, and JN3 silage exhibiting the highest abundance of *Leuconostocaceae*, *Weissella*, *Alphaproteobacteria*, *Rhizobiales*, *Rhizobiaceae*, *Enterobacterales*, *Enterobacter*, *Erwiniaceae*, and *Pantoea*, respectively (Figure 3B).

## 4. Discussion

Forage scarcity, especially of nutrient-dense green fodder, has become a critical constraint to sustainable livestock production in China. As an alternative to corn silage with equivalent agronomic performance and metabolizable energy [2], whole-plant sorghum silage offers a viable solution for alleviating this challenge, particularly in regions where rainfall is relatively low or where frequent dry periods occur such as China’s arid northwestern. Sorghum is cultivated for multiple uses, both forage- and energy-type cultivars have been developed for extended vegetative growth to maximize biomass production, while grain-type sorghum prioritizes grain yield. When selecting a cultivar for silage production, both biomass yield and nutritive value should be evaluated to optimize animal performance. In the present study, the DM biomass production of forage-type cultivar LT1 was 1.17-, 1.23-, and 1.80-fold higher than that of the grain-type cultivars JZ1531, JN3, and JZ2001, respectively. These findings are consistent with previous reports [25,26], further supporting the existing theory that genetic background may have a substantial impact on actual yield [7]. Despite its superior vegetative growth, marked by leading values in height, fresh weight, leaf number, and biomass yield, cultivar LT1 demonstrated the poorest reproductive performance, with the lowest panicle weight and proportion. The undesirable low panicle proportion observed in LT1 suggests a limitation in grain yield and starch accumulation [5]. Consequently, compared to grain-type cultivars, the silage produced from cultivar LT1 may possess lower nutritional value, potentially impairing animal performance. Furthermore, the low panicle proportion of LT1 was associated with a high moisture content (72.33%) at harvest, as the grain component typically has a lower moisture content than the vegetative parts [25]. This elevated level exceeds the 65–70% range considered optimal for high-quality silage production, potentially leading to aberrant fermentation and increased effluent and nutrient losses [9]. This was evidenced in our study, as LT1 silage exhibited the highest DM loss (1.39%) among the cultivars evaluated. Therefore, wilting prior to ensiling is recommended for forage-type cultivar LT1 to reduce moisture concentration, thereby improving fermentation quality and mitigating seepage losses. Moreover, among the grain-type cultivars, JN3 and JZ2001 outperformed JZ1531 in biomass yield.

The nutritive value of sorghum silage, which depends on chemical composition, is another critical selection criterion. Typically, grain-type sorghum exhibits a higher grain yield potential, whereas forage-type cultivars prioritize vegetative biomass, often at the expense of seed set and overall nutrient density [7]. Consistent with this expectation, silage produced from the forage-type cultivar LT1 in our study possessed the lowest nutritional density (starch, crude protein, ether extract, DM) yet the highest fiber content (NDF, ADF) among all evaluated cultivars. This nutritional profile resulted in LT1’s inferior overall nutritive value, as confirmed by its minimal total digestible nutrients (TDN) and relative feed value (RFV). Our complementary research on these same cultivars has confirmed significant differences in in vitro digestibility, with LT1 demonstrating inferior DM effective degradability (58.35% vs. 59.74–64.55% in grain-type silage) and gas production kinetics [27]. These findings corroborate the principle established by Pupo et al. [28] that yield-focused production can compromise forage nutritional quality. From a practical perspective, silage with higher starch and CP content can reduce the need for cereal grains inclusion in ruminant diets or increase dietary nutrients density. Reducing cereal grain input is particularly vital during periods of high grain prices, while enhanced dietary nutrient density is positively correlated with animal performance [5]. Notably, LT1 silage had the lowest tannin content, likely attributable to its forage-type genetic background which prioritizes biomass over grain production. Sorghum accumulates tannins in its seeds, particularly in certain bird-resistant and brown-seeded varieties [29]. Tannins are traditionally considered anti-nutritional factors in sorghum silage and grain, due to their propensity to hinder the digestion and absorption of starch and protein via cross-linking interactions with endosperm proteins and starch granules [30]. Nevertheless, their physiological impact on herbivores is fundamentally dose-dependent. When present at moderate concentrations, tannins can mitigate methane emissions from enteric fermentation, thereby concurrently reducing the risk of ruminal bloat and improving animal productivity [7]. Consequently, although the minimal tannin content of LT1 silage may confer a potential advantage in nutrient digestibility over grain-type cultivars, this potential benefit appears to be physiologically negligible in herbivores.

Among the grain-type cultivars evaluated, JN3 and JZ2001 exhibited comparable chemical composition and nutritional value, yet both of which were significantly superior to JZ1531. Notably, JN3 silage contained significantly lower levels of ADL and tannins relative to other grain-type cultivars. As lignin is a primary constraint on forage digestibility, this reduction likely enhances the bioavailability of JN3 silage, potentially leading to greater nutrient utilization efficiency in ruminants [7]. The lower lignin and tannin levels in JN3 silage, along with its distinct fermentation profile, provide a mechanistic explanation for the enhanced digestibility characteristics documented in our complementary research [27]. Coupled with JN3’s superior biomass yield—which was comparable to JZ1531 and significantly greater than JZ2001—these attributes position JN3 as the most suitable and high-performing cultivar for whole-plant sorghum silage production in northwestern China.

As is well known, ensiling is a common method for preserving high-moisture forage through LAB-mediated anaerobic fermentation. In this process, LAB convert water-soluble carbohydrates into organic acids (mainly lactate), thereby reducing pH and suppressing the development of undesirable microorganisms including clostridia, molds, and yeasts [9]. Silage quality is largely determined by the physicochemical characteristics of the raw material, including moisture content, water-soluble carbohydrate concentration, buffering capacity, and epiphytic microbial composition [31,32]. Consequently, variation in chemical composition among sorghum varieties may directly influence the ensiling fermentation process and ultimately final silage quality. Generally, all evaluated cultivars successfully produced well-ensiled whole-plant sorghum silage in this study, as evidenced by favorable fermentation characteristics: low pH (3.99–4.15), minimal ammonia-nitrogen (0.13–0.24 g/kg) and butyrate (0.87–1.17 g/kg) content, abundant organic acid content (29.79–34.99 g/kg), and a normal lactic-to-acetic acids ratio (2.19–2.91) [9]. Furthermore, the comparable microbial populations—encompassing LAB, coliform bacteria, yeasts, and molds—across all cultivars suggest that the epiphytic microbiota on the fresh plant was sufficiently abundant to initiate vigorous fermentation, ultimately yielding well-preserved silage. Nevertheless, significant cultivar-dependent differences were identified in several key fermentation parameters.

Compared to grain-type cultivars, silage from the forage-type cultivar LT1 exhibited lower pH, concomitant with reduced concentrations of total organic acids, lactate, butyrate, and valerate, and a lower lactate-to-acetate ratio. The observed reduction in pH despite lower lactate levels presents a paradox. According to the established principle, the final pH of silage is determined primarily by the lactic acid concentration and the forage’s inherent buffering capacity, the latter being positively correlated with its content of nitrogenous compounds and ash [9]. Therefore, this paradox can be attributed to LT1’s low CP content (4.5% DM) and theoretically elevated soluble nitrogen fraction—traits associated with its superior vegetative growth and low panicle proportion. We propose that this unique nitrogen profile resulted in a lower buffering capacity, facilitating more efficient acidification during ensiling process and ultimately accounting for the unexpectedly low pH and lactate-to-acetate ratio. Furthermore, this same profile also explains the significantly high ammonia nitrogen proportion of total nitrogen (NH_3_-N/TN) in LT1 silage.

As to the grain-type cultivars, JZ1531 and JZ2001 silages exhibited generally comparable fermentation profiles. However, both silages contained higher lactic acid concentrations but lower levels of formate, butyrate, valerate, and NH_3_-N, as well as a reduced NH_3_-N/TN ratio, compared to JN3 silage. As previously discussed, these observed patterns may be explained by JN3’s substantially greater CP content and its consequently enhanced buffering capacity. A high buffering capacity resists pH decline, thereby potentially favoring the survival and metabolic activity of clostridial organisms. These diverse organisms are capable of fermenting sugars to butyrate, converting lactate to butyrate, and hydrolyzing proteins to NH_3_-N [31], which ultimately resulted in elevated butyrate and NH_3_-N levels but reduced lactate concentration in JN3 silage. It is noteworthy that the butyrate concentration in JN3 silage (0.117% DM) falls within the normal range (<0.5–1.0% DM) reported for grass and legume silages [9], indicating that these levels represent expected background variation rather than abnormal fermentation. Although valerate concentrations (3.43 g/kg DM) are less frequently documented in the literature, their presence at this level reflects active secondary fermentation processes. Although the elevated clostridial activity is generally undesirable in silage production, all key fermentation parameters for JN3 silage in this study remained within established normal ranges, indicating that its overall fermentation quality was not compromised.

Ensiling is a complex microbiological process. The resultant microbial community structure and fermentation dynamics are fundamentally shaped by the metabolite profile of the raw material [33]. Consequently, profiling the silage microbiome is essential for understanding the biological mechanisms underlying ensiling and the determinants of final silage quality across different feedstocks. This study utilized amplicon sequencing to evaluate bacterial diversity in whole-plant sorghum silage from multiple cultivars. Differential analysis based on alpha diversity indices revealed that JN3 silage possessed significantly greater bacterial community richness and evenness than JZ2001 and JZ1531 silages, while LT1 silage displayed intermediate values. The diminished bacterial community richness observed in JZ2001 and JZ1531 silages may be attributed to their elevated tannin concentrations. This inference is supported by the well-documented bioactivity of sorghum tannins, which possess multifunctional properties—including antimicrobial, antioxidant, and anti-inflammatory activities—that can markedly alter the microbial ecology of the ensiling environments [34]. Furthermore, the reduced bacterial richness in LT1 silage relative to JN3 is likely due to its more rapid acidification and lower final pH, which stabilizes silage fermentation and shapes the microbial community structure by inhibiting or eliminating pH-sensitive microorganisms.

Furthermore, principal coordinate analysis based on both unweighted and weighted UniFrac metrics revealed significant differences in bacterial community structure among LT1, JN3, and JZ2001 silages. These structural differences would necessarily be accompanied by corresponding changes in metabolic profiles, ultimately leading to the variations in fermentation parameters observed among these cultivars. In contrast, while confidence ellipses indicated a potential separation trend, JZ1531’s bacterial community structure remained statistically indistinguishable from those of JN3 and JZ2001. These findings were further confirmed by LEfse analysis, which identified multiple significantly enriched bacterial taxa uniquely in LT1, JN3, and JZ2001 silages, with no significant enrichments detected in JZ1531 silage.

To further investigate cultivar-specific differences in the silage bacterial profile, we analyzed the bacterial composition at both the phylum and genus levels. Consistent with previous study [16], *Firmicutes*, *Cyanobacteria*, and *Proteobacteria* were the three dominant phyla in mature sorghum silages in this study, while *Lactobacillus*, *Chloroplast*, and *Leuconostoc* were the most abundant genera. As the predominant phylum, *Firmicutes* was primarily composed of *Lactobacillus*, *Leuconostoc*, *Weissella*, *Lactococcus*, and *Enterococcus* (Supplementary Table S1). Most of these genera are involved in lactic acid fermentation during ensiling, as established by Phalow et al. [35]. The dominance of these LAB is a well-established indicator of successful fermentation [35], which contributed to the observation that silages from all cultivars in this study were well-ensiled. Notably, taxonomic analysis revealed that 43 bacterial taxa common across all cultivar silages constituted 97.59% of the total sequences, indicating a core microbiome in well-fermented sorghum silage despite cultivar-dependent variations in community composition, as established by Henderson et al. [36].

It is reported that silage fermentation is initiated by plant-associated LAB, including species of *Lactobacillus*, *Enterococcus*, *Pediococcus*, *Leuconostoc*, *Lactococcus*, *Weissella*, and *Streptococcus* [35]. These pioneers acidify the environment, facilitating the subsequent dominance of more acid-tolerant species like *Lactobacillus plantarum* and *L. buchneri*. The makeup of this initial LAB community can vary depending on the plant species, possibly reflecting differences in the nutritional preferences of the bacteria involved. Our analyses (LEfSe and differential abundance) revealed a distinct divergence in LAB communities between sorghum types. LT1 silage was dominated by *Lactobacillus* and *Lactococcus*, whereas grain-type cultivar silages were enriched in *Leuconostoc* and *Weissella*. The LAB profile in grain-type cultivar silages was consistent with our previous finding of a *Pantoea*-to-*Leuconostoc*-to-*Lactobacillus* succession in JN3 sorghum stalk silage [16]. In contrast, the profile in LT1 silage aligned more closely with that reported for sweet sorghum silage [37,38], where the fermentation process was initially dominated by *Lactococcus*, *Weissella*, and *Pediococcus* before *Lactobacillus* became dominant. These results lend further support to a recent meta-analysis by Ridwan et al. [33], which demonstrated that the silage microbiome (particularly the LAB profile) is highly dependent on the raw material type used for the manufacture of silage. We hypothesize that this divergence stems from differences in phenolic compound content between sorghum types, coupled with the notable resilience of *Leuconostoc* species to these compounds in grain-type cultivars. These insights thus not only provide a mechanistic understanding but also directly inform a practical application: enhancing grain-type sorghum silage quality through the development of specialized lactic acid bacterial inoculants containing *Leuconostoc* spp.

Among the grain-type cultivars, JN3 silage had the most abundant *Weissella*, *Rhizobiaceae*, *Enterobacter*, and *Pantoea*, all of which are known to be primarily active in the early stages of silage fermentation [16,38]. The elevated prevalence of these taxa in the mature silage implies a prolonged anaerobic fermentation phase. This extended period likely facilitated nutrient competition between facultative or obligate anaerobic microorganisms and lactic acid bacteria, ultimately resulting in impaired fermentation characterized by increased proteolysis, attenuated acidification, and reduced lactate accumulation [9,35]. Consequently, these mechanistic insights readily account for the previously observed elevated bacterial richness, more complex fermentation end-products profile, and reduced lactic acid concentration in JN3 silage. Furthermore, the observed higher relative abundance of *Cyanobacteria* (which exist primarily as chloroplasts) in grain-type sorghum silages, especially JZ2001, compared to LT1, is likely a consequence of reduced bacterial biomass. We hypothesize that this apparent reduction is driven by the antimicrobial activity of sorghum phenolic compounds, which are more abundant in these cultivars and inhibit bacterial proliferation. The decrease in bacterial DNA effectively inflates the proportional representation of chloroplast-derived DNA in the metagenomic profile.

In summary, despite the forage-type cultivar LT1 demonstrated superior biomass yield, its low panicle proportion resulted in undesirable nutritional characteristics—specifically, high fiber, low starch and crude protein content, and inferior energy value—as well as elevated moisture at harvest that led to higher dry matter loss and risk of effluent. In contrast, JN3 achieved an optimal balance between yield and quality, exhibiting robust biomass production alongside high nutritional values and favorable fermentation parameters. Although the grain-type cultivar JN3 silage showed moderately elevated undesirable microbial activity and ammonia nitrogen, likely due to its high crude protein content and buffering capacity, all key fermentation indicators remained within acceptable ranges. Its lower lignin and tannin content further suggests enhanced digestibility and nutrient availability. The distinct microbial community structure observed in JN3 silage, characterized by early-colonizing taxa and a prolonged fermentation phase, aligns with its chemical composition and provides a microbiological basis for its fermentation profile.

## 5. Conclusions

Based on the comprehensive evaluation, JN3 represents the most promising candidate cultivar for whole-plant sorghum silage production in semi-arid northwestern China. The documented divergence in silage bacterial composition among cultivars offers a strategy for enhancing phenolics-enriched whole-plant sorghum silage quality through development of specialized inoculants containing *Leuconostoc* species. It should be noted that our findings are based on a single growing season and location under controlled experimental conditions using laboratory-scale silos. Validation through multi-location trials across multiple seasons, along with farm-scale studies and animal feeding experiments, would strengthen the practical applicability of our findings. Furthermore, while we identified key microbial patterns, future research should explore the specific mechanisms by which plant phenolic compounds modulate microbial succession during ensiling, and investigate the synergistic effects of combining *Leuconostoc*-based inoculants with other lactic acid bacteria to optimize fermentation efficiency across diverse sorghum cultivars. Notwithstanding these limitations, our findings underscore the critical importance of holistic cultivar selection based on integrated assessment of agronomic, nutritional, and microbial parameters to optimize silage quality and support sustainable livestock production.

## Figures and Tables

**Table 1:** Agronomic characteristics of four sorghum cultivars.

Items	Cultivars				SEM	*p*-Value
	JN3	LT1	JZ2001	JZ1531		
Plant height, m	1.66 ^c^	3.50 ^a^	1.51 ^d^	1.85 ^b^	0.183	<0.01
Ear height, m	1.29 ^c^	3.28 ^a^	1.17 ^c^	1.47 ^b^	0.195	<0.01
Leaf counts, Piece	10.50 ^b^	16.38 ^a^	9.50 ^b^	10.90 ^b^	0.645	<0.01
Fresh plant weight, kg	0.61 ^b^	0.96 ^a^	0.37 ^c^	0.56 ^b^	0.055	<0.01
Fresh panicle weight, kg	0.26 ^a^	0.04 ^c^	0.16 ^b^	0.21 ^a^	0.021	<0.01
Panicle proportion of whole-plant, %	42.55 ^ab^	4.16 ^c^	45.28 ^a^	39.35 ^b^	0.039	<0.01
Whole-plant fresh yield, t/ha	75.75 ^b^	118.95 ^a^	44.55 ^c^	70.35 ^b^	6.375	<0.01
Whole-plant DM yield, t/ha	26.77 ^b^	32.91 ^a^	18.29 ^c^	28.14 ^b^	1.155	<0.01

^a–d^ Means with different superscript letters within the same row differ significantly (*p* < 0.05) according to Duncan’s multiple range test; SEM, standard error of means.

**Table 2:** Chemical composition and nutritive value of whole-plant sorghum silage across various cultivars (% DM).

Items	Cultivars				SEM	*p*-Value
	JN3	LT1	JZ2001	JZ1531		
Dry matter losses	0.92 ^c^	1.39 ^a^	0.91 ^c^	1.23 ^b^	0.052	<0.01
Dry matter	37.96 ^a^	24.85 ^b^	38.56 ^a^	38.83 ^a^	1.396	<0.01
Gross energy, MJ/kg	16.78	16.88	16.82	16.82	0.020	0.30
Starch	24.30 ^a^	10.94 ^d^	21.74 ^b^	18.10 ^c^	1.278	<0.01
Crude protein	7.50 ^a^	4.50 ^d^	7.04 ^b^	5.62 ^c^	0.298	<0.01
Ether extract	1.47 ^b^	0.91 ^c^	1.68 ^a^	1.51 ^b^	0.074	<0.01
Neutral detergent fiber	29.68 ^c^	47.77 ^a^	29.21 ^c^	32.35 ^b^	1.946	<0.01
Acid detergent fiber	17.21 ^c^	28.98 ^a^	17.62 ^c^	19.64 ^b^	1.226	<0.01
Acid detergent lignin	2.43 ^c^	3.84 ^a^	3.30 ^b^	3.12 ^b^	0.131	<0.01
Ash	5.91	5.57	6.03	5.84	0.327	0.18
Tannin	0.096 ^b^	0.050 ^c^	0.154 ^a^	0.178 ^a^	0.014	<0.01
Calcium	0.23	0.23	0.24	0.21	0.006	0.46
Phosphorus	0.33 ^c^	0.17 ^d^	0.43 ^a^	0.41 ^b^	0.025	<0.01
Total digestible nutrients	75.79 ^a^	67.55 ^c^	75.51 ^a^	74.09 ^b^	0.816	<0.01
Relative feed value	237.00 ^a^	129.75 ^c^	240.44 ^a^	212.44 ^b^	11.035	<0.01

^a–d^ Means with different superscript letters within the same row differ significantly (*p* < 0.05) according to Duncan’s multiple range test; SEM, standard error of means.

**Table 3:** Fermentation parameters and microbial counts in whole-plant sorghum silage across various cultivars.

Items	Cultivars				SEM	*p*-Value
	JN3	LT1	JZ2001	JZ1531		
Fermentation parameters						
pH	4.12 ^a^	3.99 ^b^	4.15 ^a^	4.11 ^a^	0.013	<0.01
NH_3_-N, g/kg	0.24 ^a^	0.13 ^c^	0.18 ^b^	0.14 ^c^	0.010	<0.01
NH_3_-N/TN, %	5.29 ^b^	7.32 ^a^	4.24 ^c^	4.03 ^c^	0.314	<0.01
Total organic acids, g/kg	34.99 ^a^	29.79 ^b^	33.85 ^a^	34.08 ^a^	0.533	<0.01
Lactate, g/kg	21.22 ^b^	17.13 ^c^	23.26 ^a^	23.61 ^a^	0.620	<0.01
Formate, g/kg	1.48 ^a^	1.51 ^a^	0.74 ^b^	0.60 ^b^	0.103	<0.01
Acetate, g/kg	7.67	7.83	7.84	8.23	0.109	0.31
Butyrate, g/kg	1.17 ^a^	0.87 ^c^	1.00 ^b^	0.88 ^c^	0.031	<0.01
Valerate, g/kg	3.43 ^a^	2.59 ^b^	1.01 ^c^	0.76 ^c^	0.272	<0.01
Lactate/Acetate	2.78 ^a^	2.19 ^b^	2.97 ^a^	2.81 ^a^	0.081	<0.01
Microbial counts, lg cfu/g FM						
Lactic acid bacteria	6.79	6.62	7.01	7.06	0.060	0.09
Coliform bacteria	4.10	4.31	4.26	4.36	0.039	0.09
Yeasts	4.62	4.54	4.60	4.54	0.028	0.53
Molds	2.08	2.41	<2.00	2.60	–	–

^a–c^ Means with different superscript letters within the same row differ significantly (*p* < 0.05) according to Duncan’s multiple range test; SEM, standard error of means; –, not available.

**Table 4:** Alpha diversity indices of bacterial microbiota in whole-plant sorghum silage across various cultivars.

Items	Cultivars				SEM	*p*-Value
	JN3	LT1	JZ2001	JZ1531		
Observed features	248 ^a^	212 ^ab^	182 ^b^	153 ^b^	11.0	0.01
Shannon entropy	4.21 ^a^	3.76 ^ab^	3.33 ^b^	3.26 ^b^	0.136	0.02
Pielou’s evenness	0.53 ^a^	0.49 ^b^	0.45 ^b^	0.45 ^b^	0.014	0.03
Faith PD	15.02	15.32	12.78	12.50	0.621	0.23

^a, b^ Means with different superscript letters within the same row differ significantly (*p* < 0.05) according to Kruskal–Wallis (pairwise) test; SEM, standard error of means.

## Data Availability

The raw amplicon sequencing data were deposited into the Sequence Read Archive (SRA) of NCBI database and can be accessed via accession number PRJNA1304748.

## Supplementary Materials

The following supporting information can be downloaded at: https://www.mdpi.com/article/10.3390/microorganisms13112634/s1, Figure S1: Alpha rarefaction plots based on Shannon entropy (left) and Faith phylogenetic diversity (right) for each sample; Table S1: The detected bacterial taxa at phylum and genus levels in the whole-plant sorghum silage across cultivars.

## Abbreviations

The following abbreviations are used in this manuscript:

ADF	Acid detergent fiber
ADL	Acid detergent lignin
ASVs	Amplicon sequence variants
CP	Crude protein
DM	Dry matter
EE	Ether extract
GE	Gross energy
GLM	General linear model
LAB	Lactic acid bacteria
LDA	Linear discriminant analysis
LEfSe	Linear discriminant analysis effect size
NDF	Neutral detergent fiber
NFC	Non-fiber carbohydrates
NH_3_-N	Ammoniacal nitrogen
PD	Phylogenetic diversity
PERMANOVA	Permutational multivariate analysis of variance
RFV	Relative feed value
TDN	Total digestible nutrients
