# Supplementary material for: Cultivar-Dependent Differences in Agronomic Characteristics, Nutritional Value, Fermentation Quality, and Bacteriome Profile of Whole-Plant Sorghum Silage
Source: Microorganisms. 2025 Nov 20;13(11):2634. doi: 10.3390/microorganisms13112634 (PMC12654530; doi:10.3390/microorganisms13112634)
Supplement: Supplementary file 1 [file microorganisms-13-02634-s001.zip › microorganisms-3987531-supplementary.pdf]

Supplementary Material

Supplementary Table S1. The detected bacterial taxa at phylum and genus levels in the whole-plant sorghum silage across cultivars. %

Phylum/Genus	JN3	JZ1531	JZ2001	LT1	SEM	*P*-value
Firmicutes	50.21	56.01	39.52	65.01	3.777	0.10
Lactobacillus	29.19b	38.63ab	21.42b	54.75a	3.962	<0.01
Leuconostoc	18.43a	15.16ab	16.10ab	8.33b	1.498	<0.05
Weissella	2.27a	1.53a	1.64a	0.09b	0.235	<0.01
Lactococcus	0.06b	0.03b	0.02b	0.44a	0.063	0.02
Enterococcus	0.03	0.16	0.12	0.20	0.048	0.65
Romboutsia	0.00	0.04	0.01	0.18	0.034	0.19
Clostridium sensu stricto 1	0.01	0.08	0.02	0.11	0.019	0.18
Cyanobacteria	22.81ab	24.83ab	39.47a	8.47b	3.947	0.03
Chloroplast	22.81ab	24.83ab	39.45a	8.45b	3.918	0.03
Proteobacteria	23.92	17.20	19.23	19.74	1.042	0.09
Achromobacter	2.99	6.12	5.99	5.93	0.586	0.15
Mitochondria	3.48	3.35	4.45	1.84	0.462	0.27
Sphingomonas	3.15	2.54	2.61	2.91	0.165	0.56
Pantoea	2.48a	0.84b	0.59b	1.18b	0.205	<0.01
Methylobacterium	1.18b	0.82ab	1.09ab	0.25b	0.108	<0.01
Rhizobium	1.35a	0.38b	0.63b	0.27b	0.119	<0.01
Enterobacteriaceae	1.42a	0.04c	0.62b	0.40bc	0.134	<0.01
Enterobacter	1.93a	0.01b	0.06b	0.13b	0.208	<0.01
Aureimonas	0.97a	0.44bc	0.62b	0.14c	0.089	<0.01
Massilia	0.47b	0.26b	0.12b	1.12a	0.11	<0.01
Paraburkholderia	0.23	0.57	0.43	0.71	0.074	0.09
Alcaligenaceae	0.00	0.07	0.00	1.78	0.312	0.08
Pseudomonas	0.23b	0.16b	0.05b	0.58a	0.063	<0.01
Serratia	0.27a	0.03b	0.27a	0.37a	0.040	<0.01
Ralstonia	0.12	0.29	0.16	0.19	0.035	0.34
Kosakonia	0.45	0.03	0.08	0.12	0.071	0.13
Brevundimonas	0.34a	0.06c	0.18b	0.10bc	0.031	<0.01
Rahnella1	0.56a	0.05b	0.02b	0.00b	0.063	<0.01
Devosia	0.35a	0.07bc	0.14b	0.02c	0.034	<0.01
Hafnia-Obesumbacterium	0.22a	0.00b	0.16a	0.17a	0.03	0.03
Acinetobacter	0.14	0.10	0.06	0.23	0.024	0.07
Roseomonas	0.21a	0.16a	0.10ab	0.04b	0.024	0.03
Comamonadaceae	0.14	0.07	0.07	0.11	0.014	0.30

Phylum/Genus	JN3	JZ1531	JZ2001	LT1	SEM	*P*-value
Variovorax	0.07	0.09	0.04	0.12	0.013	0.16
Klebsiella	0.20a	0.00b	0.03b	0.04b	0.022	<0.01
Stenotrophomonas	0.13a	0.05b	0.02b	0.02b	0.016	<0.01
Paracoccus	0.09	0.06	0.04	0.02	0.018	0.60
Spirochaetota	0.08	0.10	0.01	4.55	0.817	0.09
Brevinema	0.08	0.10	0.01	4.54	0.814	0.09
Actinobacteriota	1.70a	0.82b	0.95b	0.48c	0.120	<0.01
Microbacteriaceae	0.48a	0.12b	0.16b	0.09b	0.042	<0.01
Curtobacterium	0.28a	0.28a	0.11b	0.01b	0.034	<0.01
Quadrisphaera	0.11a	0.06ab	0.04b	0.00b	0.013	<0.01
Bacteroidota	1.02	0.80	0.33	0.98	0.104	0.07
Chryseobacterium	0.35a	0.21ab	0.06b	0.16ab	0.037	0.04
Pedobacter	0.30	0.26	0.07	0.11	0.042	0.14
Hymenobacter	0.03b	0.05b	0.02b	0.13a	0.012	<0.01
Deinococcota	0.03b	0.03b	0.03b	0.17a	0.017	<0.01
Deinococcus	0.03b	0.03b	0.02b	0.17a	0.018	<0.01
Myxococcota	0.03b	0.02b	0.07ab	0.11a	0.014	<0.05
Acidobacteriota	0.03	0.01	0.10	0.06	0.016	0.21
Chloroflexi	0.02	0.01	0.06	0.03	0.010	0.18
Verrucomicrobiota	0.01	0.03	0.04	0.01	0.008	0.38
Bdellovibrionota	0.03	0.01	0.01	0.01	0.004	0.06
Planctomycetota	0.03	0.01	0.01	0.02	0.006	0.48
Patescibacteria	0.01	0.02	0.01	0.00	0.003	0.28
Others	0.06	0.11	0.15	0.36	0.068	0.38

Note: Means with different letters within the same row had significant difference ($P<0.05$)

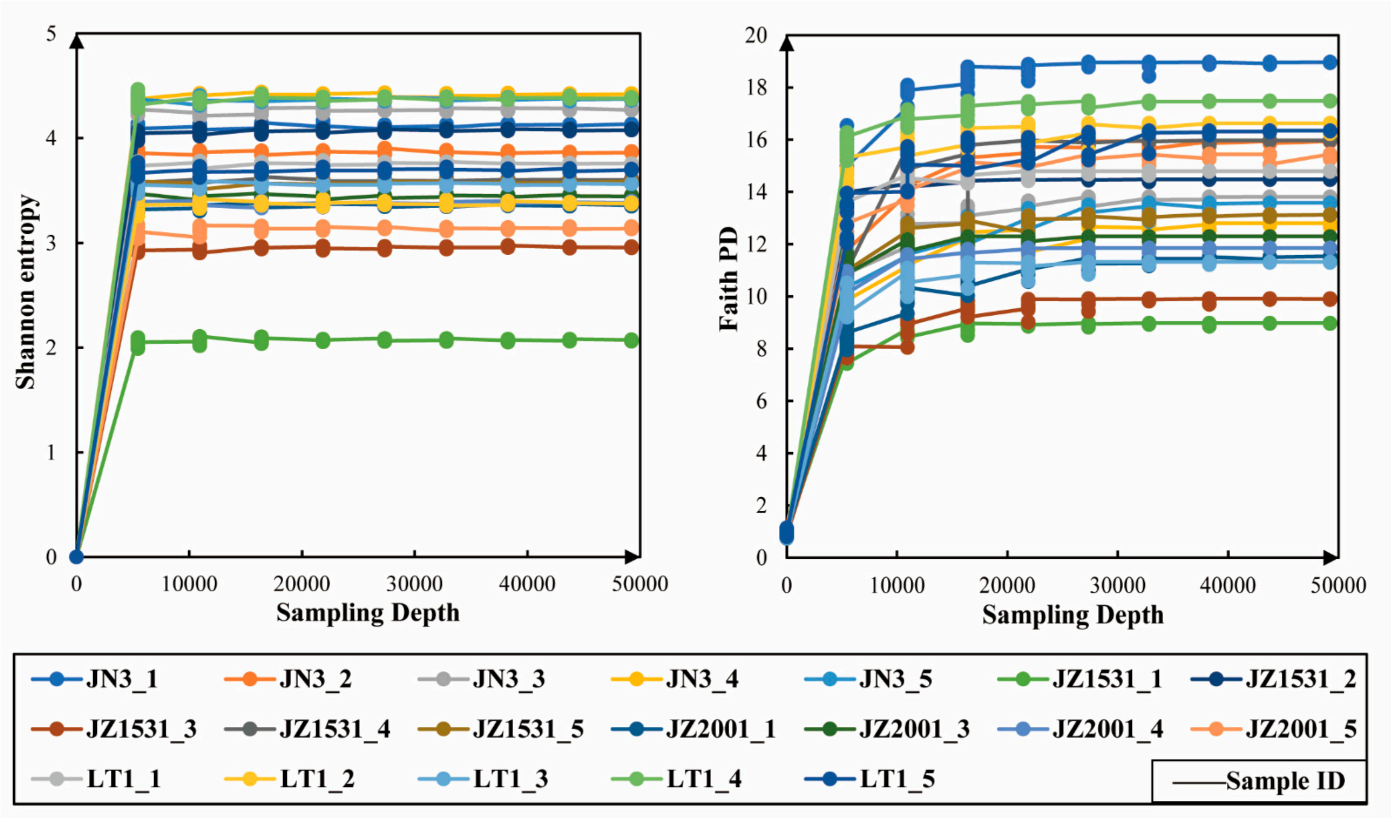

Supplementary Figure S1. Alpha rarefaction plots based on Shannon entropy (left) and Faith phylogenetic diversity (right) for each sample.
